# *Notes from the Field:* Nitazene-Related Deaths — Tennessee, 2019–2021

**DOI:** 10.15585/mmwr.mm7137a5

**Published:** 2022-09-16

**Authors:** Allison Roberts, Jessica Korona-Bailey, Sutapa Mukhopadhyay

**Affiliations:** 1 Office of Informatics and Analytics, Tennessee Department of Health.

Nitazenes are a novel group of powerful illicit synthetic opioids derived from 2-benzylbenzimidazole that have been linked to overdose deaths in several states (*1*). Nitazenes were created as a potential pain reliever medication nearly 60 years ago but have never been approved for use in the United States (*2*). Laboratory test results indicate that the potency of certain nitazene analogs (e.g., isotonitazene, protonitazene, and etonitazene) greatly exceeds that of fentanyl, whereas the potency of the analog metonitazene is similar to fentanyl (*3*,*4*). Naloxone has been effective in reversing nitazene-involved overdoses, but multiple doses might be needed (*3*,*4*). The prevalence of nitazene deaths in the United States is unknown and the frequency of nitazene involvement in overdose deaths in Tennessee has not yet been assessed. However, of concern is that nitazenes are increasingly recorded in toxicology reports and death certificate cause-of-death fields. Given their potency, raising awareness about nitazenes and implementing strategies to reduce harm through increased testing, surveillance, and linkage to treatment for substance use disorders are of vital importance. 

The Office of Informatics and Analytics at the Tennessee Department of Health conducts routine surveillance of fatal drug overdoses using the Tennessee State Unintentional Drug Overdose Reporting System (TN SUDORS). The surveillance system collects sociodemographic information and circumstances associated with overdose deaths, including death scene information, autopsy reports, and toxicology reports for drug overdose deaths of unintentional and undetermined intent. For this analysis, nitazene-involved deaths were identified using a text search for the term “nitazene” (and common misspellings) in death certificate cause-of-death fields and in toxicology reports for deaths that occurred during January 1, 2019–December 31, 2021, with data available as of June 10, 2022. TN SUDORS data were examined for demographic characteristics and circumstances surrounding deaths. Tennessee death certificate data for 2021 are provisional, as are SUDORS data for July–December 2021. This analysis was determined to be exempt from review by the Tennessee Department of Health’s Institutional Review Board and was reviewed by CDC and conducted consistent with applicable federal law and CDC policy.*

During 2019–2021, a total of 52 nitazene-involved fatal drug overdoses were identified using TN SUDORS data, including no cases in 2019, 10 in 2020, and 42 in 2021 (Table). In 2020, most of nitazene-involved deaths were attributed to isotonitazene, but in 2021, most were attributed to metonitazene. Among the 10 nitazene-involved overdose deaths identified in 2020, the average decedent age was 40.6 years, and nine (90.0%) decedents were White males. In 2021, the average decedent age was similar (42.6 years); a smaller percentage of the 42 decedents were male (66.7%) and White (88.1%). Whereas nitazene-involved deaths increased in 2021, 85.7% were attributed to metonitazene, which has a lower potency compared with other nitazenes. All nitazene-involved overdoses involved multiple substances. During both 2020 and 2021, the most frequent route of administration was injection (18; 34.6%). Other routes of administration were smoking, snorting, and ingestion. In addition to fentanyl (59.6%), other co-occurring substances included methamphetamine (46.2%), amphetamine (25.0%), and flualprazolam (13.5%).

**TABLE:** Demographic characteristics of nitazene-involved overdose deaths (N = 52) — Tennessee State Unintentional Drug Overdose Reporting System,* 2020–2021

Characteristic	No. (%)	
	2020	2021
**Total**	10 (100.0)	42 (100.0)
**Age, yrs, mean (SD)**	40.6 (13.2)	42.6 (12.1)
**Sex**		
Female	1 (10.0)	14 (33.3)
Male	9 (90.0)	28 (66.7)
**Race**		
Other	0 (—)	5 (11.9)
White	10 (100.0)	37 (88.1)
**Nitazene^†^**		
Metonitazene	1 (10.0)	36 (85.7)
Isotonitazene	9 (90.0)	1 (2.4)
Protonitazene	0 (—)	2 (4.8)
Etonitazene	0 (—)	5 (11.9)

**Abbreviation**: SUDORS = State Unintentional Drug Overdose Reporting System.

* SUDORS deaths were identified via Tennessee Department of Health, Division of Vital Records and Statistics, Death Statistical System, 2019-2021. 2021 death statistical data are provisional.

^†^ Categories are not mutually exclusive. Nitazene analogs have differing potency. The potency of isotonitazene, protonitazene, and etonitazene greatly exceeds that of fentanyl, whereas the potency of metonitazene is similar to fentanyl.

Most nitazene-involved deaths in Tennessee were identified in Knox County. This apparent high prevalence is most likely because Knox County’s Regional Forensic Center sends blood samples for secondary laboratory testing to the Drug Enforcement Agency (DEA) (*5*); traditional laboratory panels do not always capture nitazenes. Therefore, nitazene-involved deaths that occur in other counties of Tennessee are likely to be undercounted. DEA provides laboratory testing as a free resource and encourages state and national forensic centers to submit their samples for additional testing to assist in the accurate counting of deaths and to better guide drug overdose prevention efforts.

Naloxone was only administered to 12 (23%) persons with nitazene-involved fatal overdoses. Given naloxone’s effectiveness in preventing fatal overdoses, more frequent administration of naloxone by first responders, bystanders, and clinicians is important. Implementing naloxone training and distribution efforts throughout all states is also necessary. As with fentanyl, multiple naloxone doses might be required because of the potency of nitazene^†^ and can be safely administered. In addition, contacting emergency services is necessary to provide immediate medical attention to persons who might be overdosing.

Four times as many nitazene-involved overdoses were identified in Tennessee in 2021 than in 2020, and this number could be underestimated because of low testing frequency. Nitazenes are an emerging group of highly potent psychoactive substances, tests for which are often not included in standard toxicology panels. Given their potency, raising awareness about nitazenes and implementing strategies to reduce harm through increased testing, surveillance, and linkage to treatment for substance use disorders are of vital importance. More data are required to better understand this emerging group of psychoactive substances in the United States.

## Footnotes

* 45 C.F.R. part 46, 21 C.F.R. part 56; 42 U.S.C. Sect. 241(d); 5 U.S.C. Sect. 552a; 44 U.S.C. Sect. 3501 et seq.

†
https://health.usnews.com/drugs/articles/nitazenes

## References

[1] Vandeputte MM, Van Uytfanghe K, Layle NK, St Germaine DM, Iula DM, Stove CP. Synthesis, chemical characterization, and *μ*-opioid receptor activity assessment of the emerging group of “nitazene” 2-benzylbenzimidazole synthetic opioids. ACS Chem Neurosci 2021;12:1241–51. 10.1021/acschemneuro.1c00064 33759494

[2] Expert Committee on Drug Dependence. Critical review report: isotonitazene. Geneva, Switzerland: World Health Organization, Expert Committee on Drug Dependence; 2022. https://www.who.int/docs/default-source/controlled-substances/43rd-ecdd/isonitazene-43rd-final-complete-a.pdf?sfvrsn=c98d9c9_2

[3] Vandeputte MM, Krotulski AJ, Walther D, Pharmacological evaluation and forensic case series of N-pyrrolidino etonitazene (etonitazepyne), a newly emerging 2-benzylbenzimidazole ‘nitazene’ synthetic opioid. Arch Toxicol 2022;96:1845–63. 10.1007/s00204-022-03276-4 35477798 PMC12964052

[4] Public Health Ontario. Novel non-fentanyl synthetic opioids: risk assessment and implications for practice. Toronto, ON: Ontario Agency for Health Protection and Promotion, Public Health Ontario; 2021. https://www.publichealthontario.ca/-/media/documents/e/2021/evidence-brief-novel-opioids-risk-analysis-implications.pdf?sc_lang=en

[5] Diversion Control Division. DEA Tox toxicology testing program. Springfield, Virginia: US Department of Justice, Drug Enforcement Administration; 2022. Accessed May 26, 2022. https://www.deadiversion.usdoj.gov/dea_tox/index.html

